# The Endermic Method, Theoretically and Practically Considered

**Published:** 1842-10

**Authors:** 


Art. V.—*De Methodi Endermaticæ Ratione nec non applicatione.* Auctore Ferdinando Schubert, Philosophiæ et Medicinæ Doctore. —*Schaffnaburghi*, 1841, pp. 80.

*The Endermic Method, theoretically and practically considered.* By Ferdinand Schubert, Ph. & m.d.—*Schaffhausen*, 1841.

This treatise purports to give the result of two years' observation of this plan of treatment, and to fill up to a certain degree the imperfections existing in this branch of medicine. The preface is devoted to a brief outline of those who have employed the endermic method in various countries. The conditions under which this treatment can be employed with advantage are, according to the author, numerous, but from the account of the practical employment of the various remedies, this mode of treatment appears in many cases to be attended with much more pain and suffering than their internal employment.

"Symptoms accompanying the endermic application of medicines. The immediate effect of the removal of the cuticle is an irritation of the nerves of the part from the exposure to the atmosphere. The action of solid substances when uniform in their composition, depends on their roughness of surface; when dissimilar in composition on their chemical action. The immediate effect produced, which is frequently attended with great pain, is termed the local or primary effect, as distinguished from the general effect, which soon succeeds. This primary effect consists most commonly in a sensation of the most intense heat in the part, like that of burning coals, a feeling of creeping or crawling in the part, with shooting and tearing pains, the cutis subsequently inflames, suppurates, and sometimes ulcerates. . . . . In a shorter or longer period, according to the nature of the application, effects are produced in the remote organs, which are called the secondary effects, and are more or less directly connected with the primary effect. These effects resemble in their kind those resulting from the internal administration of the same medicine, and are materially increased by its application near the diseased part." (p. 33.)

The second part contains illustrations of the effects of the various medical agents, which have been employed endermically. The part devoted to the minerals is very brief, and contains very little new, and the small quantity of information derived from various sources is not of a very complete or practical nature. The part devoted to the medicines of the vegetable kingdom is more full and contains a good summary from various practitioners of the endermic method. Some of the medicines are said to have been found useful in the most serious diseases, as carcinoma and diabetes, but paralytic and nervous affections appear, from the statements of the author and the observations of others, to have been the forms of disease in which most benefit resulted from their employment.

